# Assessing the impact of tobacco smoke exposure on earlyonset osteoarthritis: A cross-sectional analysis of secondary data from the National Health and Nutrition Examination Survey (NHANES), 1999–2020

**DOI:** 10.18332/tid/208426

**Published:** 2025-09-30

**Authors:** Yanchao Li, Xiangmin Wan, Wei Long

**Affiliations:** 1 Department of Orthopedics, The First People’s Hospital of Yibin, Yibin, China; 2 School of Graduates, Dalian Medical University, Dalian, China; 3 Department of Ophthalmology, The First People’s Hospital of Yibin, Yibin, China

**Keywords:** tobacco smoke exposure, early-onset OA, serum cotinine, non-linearity, NHANES

## Abstract

**INTRODUCTION:**

There is evidence that exposure to tobacco smoke is associated to a number of chronic diseases, but the evidence for an association with osteoarthritis (OA) is sparse and inconclusive. The aim of this study was to investigate whether exposure to tobacco smoke for an adult is associated with developing OA at a young age, and to assess dose–response patterns.

**METHODS:**

We conducted a pooled, cross-sectional analysis of secondary data from the National Health and Nutrition Examination Survey (NHANES) 1999–2020 among US adults aged 20–54 years (n=26145). Tobacco smoke exposure was quantified by serum cotinine. Multivariable logistic regression, restricted cubic splines, and threshold analyses were used to estimate the dose–response relationship between cotinine and self-reported physician-diagnosed early-onset OA.

**RESULTS:**

Overall, 1086 participants (4.2%) reported early-onset OA. After full adjusted odds ratio (AOR), serum cotinine ≥3 ng/mL was associated with a 52% increase in odds of early-onset OA (AOR=1.52; 95% CI: 1.30–1.79), compared with <0.05 ng/mL. A non-linear, positively saturated relationship was observed between the cotinine levels after the natural logarithm (LN) transformation and early-onset OA, with an inflection point at approximately 2.90 ng/mL (AOR=1.38; 95 % CI: 1.17–1.63, p=0.00). Subgroup analyses confirmed the robustness of this association across demographic and clinical strata.

**CONCLUSIONS:**

This study, based on a nationally representative sample from the United States, suggests that high levels of tobacco smoke exposure significantly increase the likelihood of early-onset OA, highlighting the need for further research into factors associated with early-onset OA.

## INTRODUCTION

Osteoarthritis (OA) is a chronic condition characterized by cartilage degradation, synovial inflammation, subchondral bone remodeling, and the development of osteophytes^1^. It is a widely recognized cause of disability on a global scale and is commonly perceived as an ailment affecting older individuals^2^. However, epidemiological evidence indicates that OA increasingly affects younger and middle-aged adults^3^. Early-onset OA, defined here as diagnosis before the age of 55 years, imposes substantial economic and societal burdens, with global costs exceeding US$106.87 billion annually^3^. Identifying modifiable risk factors is therefore critical for developing preventive strategies.

Tobacco smoke exposure-encompassing both active smoking and secondhand smoke is a wellestablished risk factor for numerous chronic disorders^4^. Commercially available cigarettes release a variety of harmful substances such as tar, nicotine and nitrosamines during combustion^5^, which have been linked to thyroid dysfunction, osteoporosis, and various malignancies^6,7^. However, the relationship between tobacco smoke exposure and OA remains unclear^8^. Evidence from a multitude of clinical studies across various global populations suggests an inverse relationship between cigarette smoking and the likelihood of developing OA^9,10^. Some evidence indicates that individuals who do not smoke tend to have a higher body mass index (BMI) than smokers, contributing to a higher prevalence of OA^11,12^. In their investigation, Salis et al.^13^ reported that hip OA showed no significant link to smoking habits in terms of its baseline prevalence, incidence, or longitudinal progression over 4–5 years. However, this observed protective association has been met with skepticism within the scientific community. Critics argue that it may be an artifact of selection bias, notably bias introduced by hospital-based recruitment of participants, rather than a true biological effect^14^. Given the short half-life and instability of nicotine in the human body, it poses a challenge as an indicator for assessing tobacco smoke exposure^15^. As a superior objective indicator, cotinine – the principal metabolite of nicotine derived from tobacco combustion – provides a more dependable measure of exposure. Its extended biological half-life and consistent metabolic clearance ensure that serum cotinine concentrations serve as a precise proxy for recent tobacco smoke exposure^16^. Consequently, cotinine is extensively employed as a biomarker in scientific research to quantify tobacco-derived constituents in biological specimens. Beyond cotinine, other tobacco-related biomarkers like cadmium may offer additional mechanistic insights. Emerging evidence indicates that tobacco use constitutes a significant route of cadmium exposure, which may promote joint pathology through mechanisms such as essential element depletion, oxidative stress induction, protein citrullination, and inflammatory activation – processes implicated in the pathogenesis of osteoarthritis (OA)^17^. Thus, a multi-biomarker approach is advocated to comprehensively evaluate the health impacts of tobacco smoke. Nevertheless, direct epidemiological evidence establishing a link between tobacco smoke exposure and early-onset OA remains limited.

This study seeks to examine the association between tobacco smoke exposure and early-onset osteoarthritis, with the goal of generalizing the results to broader populations and enhancing comprehension of its pathological influence. The findings are anticipated to yield novel perspectives and contribute to formulating preventive and therapeutic approaches for early-onset OA management.

## METHODS

### Data sources and study population

**Figure 1:**
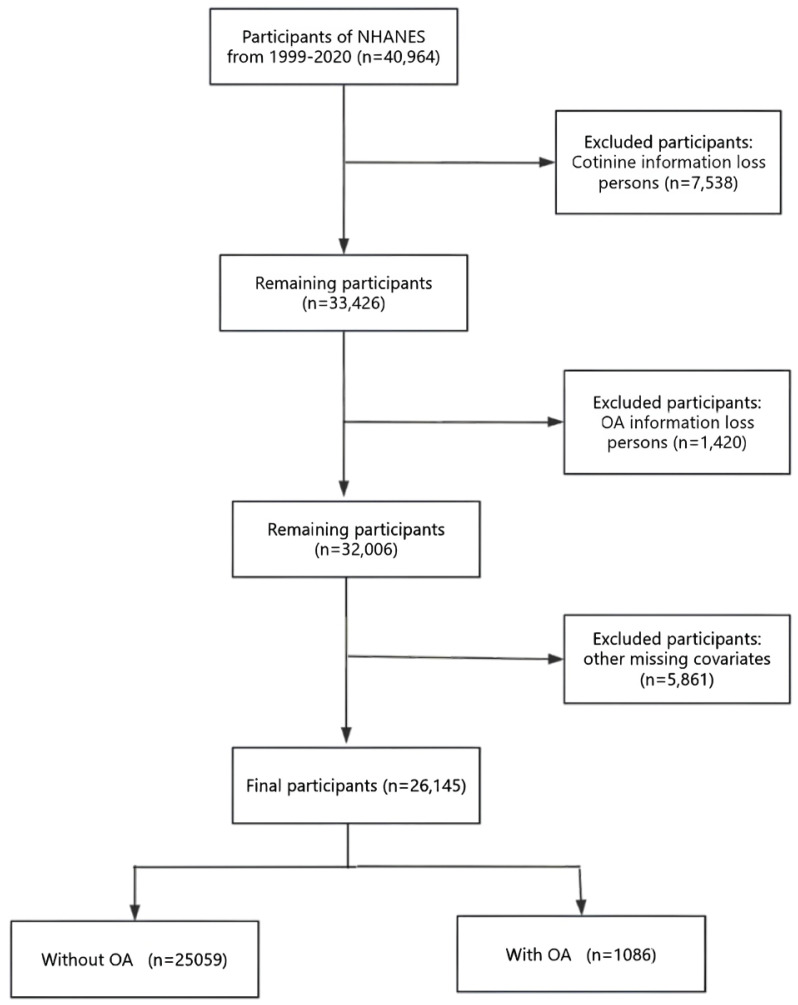
Study flow diagram for the cross-sectional analysis of smoking and early-onset OA in the NHANES, 1999–2020 (N=26145)

This study employed a pooled analysis of secondary data from the National Health and Nutrition Examination Survey (NHANES) covering the period from 1999 to 2020 among US adults aged 20–54 years (n=26145). The NHANES, coordinated by the National Center for Health Statistics (NCHS), is a comprehensive survey designed to assess the health and nutritional status of the non-institutionalized population in the United States. The survey uses a nationally representative sampling design that uses stratified, multistage probability cluster sampling to ensure a diverse and representative sample. This is achieved through standardized household interviews covering demographics, medical history, and lifestyle, as well as on-site physical examinations, laboratory tests, and anthropometric measurements among the non-institutionalized US population. The NHANES study protocols received ethical approval from the NCHS Research Ethics Review Board, and written informed consent was obtained from all participants. As this research involves secondary analysis of pre-existing data, additional approval from an Institutional Review Board was deemed unnecessary. All procedures in this study were performed in line with the principles of the Declaration of Helsinki. Furthermore, the reporting of this cross-sectional research follows the Strengthening the Reporting of Observational Studies in Epidemiology (STROBE) guidelines. We used data from eleven consecutive cycles (1999–2020) of the NHANES. Our analysis encompassed participants aged 20–54 years who had successfully completed all necessary assessments (n=40964). We excluded individuals with missing data on serum cotinine (n=7538), missing data on OA (n=1420), and other missing covariates (n=5861). The final study sample consisted of 26145 participants. The use of data from 1999 to 2020 was driven by the need for a sufficiently large sample size to ensure adequate statistical power for detecting associations between tobacco smoke exposure and early-onset OA, given the relatively low prevalence of early-onset OA in the population. The flow of participant selection is depicted in Figure 1.

### Assessment of tobacco smoke exposure

Cotinine, a metabolite of nicotine, can indicate whether a person is currently smoking, or has been exposed to tobacco smoke in the environment^18^. Serum cotinine concentrations were quantified via isotope-dilution high-performance liquid chromatography coupled with atmospheric pressure chemical ionization tandem mass spectrometry (ID HPLC-APCI MS/MS). This method demonstrates a lower limit of detection of 0.02 ng/mL and an intra-assay coefficient of variation (CV) below 10%, reflecting its high analytical precision and reliability for serum cotinine quantification.

### OA definition

Data regarding arthritis diagnoses were obtained through in-person interviews based on self-report. Participants were inquired whether a physician or healthcare provider had ever diagnosed them with arthritis. Affirmative respondents were further requested to specify their diagnosis type, selecting from OA, rheumatoid arthritis, psoriatic arthritis, or other forms. Self-reported physician-diagnosed OA represents the most prevalent case definition in epidemiological research^19^. An earlier investigation demonstrated 81% concordance between selfreported and clinically confirmed OA diagnoses^20^. For the purpose of this study, early-onset OA was defined as a diagnosis of OA before the age of 55 years. This definition is consistent with the previous epidemiological studies, which classify OA occurring before the age of 55 years as early-onset^3^. This stratification allows us to focus on the subset of the population that may be more susceptible to the effects of tobacco smoke exposure at a younger age.

### Covariables data extraction

The NHANES data collection process included the administration of a standardized questionnaire to participants through household interviews, supplemented by a comprehensive medical assessment for each individual. The selection of covariates was based on previous reports in the literature and clinical experience. The covariates included in this study were age, gender, marital status, race, education level, family income, hypertension, diabetes, coronary heart disease(CHD), body mass index (BMI, kg/m^2^), alcohol use, serum total cholesterol (TC) (mmol/L), serum direct HDL cholesterol (HDL-C) (mmol/L), serum calcium (mg/dL), serum phosphorus (mg/ dL), blood urea nitrogen (BUN) (mg/dL), and serum uric acid (UA) (mg/dL). Marital status was classified into two categories: married or living with a partner, and living alone. This categorization was chosen to reflect the social support and living conditions of the participants. Race was categorized as non-Hispanic White, non-Hispanic Black, Mexican American, or other race. These categories were selected to align with the standard racial/ethnic classifications used in NHANES and to allow for meaningful subgroup analyses. Education level was divided into three groups: <9, 9–12, and >12 years. These categories were used to assess the impact of education level on health outcomes, with <9 years indicating low education, 9–12 years indicating high school education, and >12 years indicating higher education. Family income was categorized using the poverty income ratio (PIR) into low (PIR ≤1.30), medium (1.30< PIR ≤3.50), and high (PIR >3.50) tiers, following the classification criteria established in a US government report^21^. These income categories were chosen to reflect the economic status of the participants and to allow for analysis of potential socioeconomic disparities in health outcomes. The presence of hypertension, diabetes, coronary heart disease (CHD), and stroke was determined through self-reported physician diagnoses. BMI was derived from standardized measurements of height and weight. Alcohol consumption was classified as positive if participants reported consuming a minimum of 12 drinks per year, encompassing all varieties of alcoholic beverages such as spirits, beer, wine, and wine coolers.

### Weights processing

To ensure the national representativeness of the study results, we processed sample weights in strict accordance with official NHANES guidelines. As this study integrated NHANES data from five cycles from 1999 to 2020, we used the following weight processing steps: 1) weight standardization, sample weights for each cycle were standardized using standardized weight=original weight/mean of weights for that cycle; 2) weight merging, the standardized weights were merged into a single unified merged weight = standardized weight/number of cycles; and 3) weighted analyses, all statistical analyses were performed using the merged weights to ensure that the results reflected the true picture of the US national population^22^.

### Statistical analysis

Normally distributed continuous variables are presented as mean ± standard deviation (SD), whereas non-normally distributed variables are summarized as median and interquartile range (IQR). Categorical variables are reported as frequencies and percentages, and group comparisons were performed using analysis of variance, Kruskal–Wallis tests, or chi-squared tests, as appropriate. To address the skewed distribution of serum cotinine levels, a natural logarithm (LN) transformation was applied to better approximate a normal distribution. The transformed variable was subsequently analyzed as continuous in the sensitivity analysis. In line with established literature, we categorized cotinine levels to reflect varying degrees of tobacco smoke exposure, adopting the recently updated threshold of 3 ng/mL proposed by Benowitz et al.23 to distinguish active smokers from nonsmokers. The classification was structured as follows: cotinine concentrations (ng/mL) <0.05 no exposure (non-smokers); 0.05–2.99 low exposure, consistent with secondhand smoke exposure; and ≥3 ng/mL heavy exposure, corresponding to active smoking.

In addition, to evaluate the association between tobacco smoke exposure and the risk of earlyonset OA, we computed odds ratios (OR) and corresponding 95% confidence intervals (95% CI) through multivariable logistic regression analyses. Four different models were used in this analysis. Model 1: unadjusted. Model 2 adjusted for age, gender, race, marital status, education level, and family income. Model 3 as for Model 2 plus BMI, TC, HDL-C, serum calcium, serum phosphorus, BUN and UA. Model 4 as for Model 3 plus alcohol use, hypertension, diabetes, CHD and stroke. After adjusting for all covariates included in Model 4, a multivariate-adjusted restricted cubic spline (RCS) analysis was performed to assess the potential nonlinear dose-response association between LNtransformed serum cotinine levels and early-onset OA. The number and positions of knots were determined according to the sample size and data distribution. Four knots were placed at the 5th, 35th, 65th, and 95th percentiles to achieve an optimal trade-off between model flexibility and goodness-of-fit, thereby allowing the RCS model to effectively represent potential non-linearity while avoiding overfitting. This strategy aligns with established methodological guidelines for applying RCS in large-sample studies. To examine non-linearity, the significance of the nonlinear components in the model was assessed using a Wald test. A p-value for non-linearity was derived to determine whether the dose-response relationship significantly departed from a linear pattern. A p<0.05 was considered indicative of a non-linear association. Subgroup analyses were conducted to assess whether the association between tobacco smoke exposure and early-onset OA was modified by specific variables. The variables examined included age (<40 vs ≥40 years), marital status (married/living with a partner vs living alone), BMI (<25, 25–30 vs ≥30 kg/m^2^), alcohol use, hypertension, and diabetes.

All statistical analyses were conducted using the R statistical software (http://www.R-project.org, The R Foundation) and Free Statistics software (version 2.1.1). Participant characteristics were summarized using descriptive statistics, and a twotailed significance level of p<0.05 was adopted for all hypothesis tests.

## RESULTS

### Basic characteristics

The baseline demographic and clinical profiles of the study participants are presented in Table 1. Overall, among the 26145 individuals, 1086 (4.20%) had OA. The mean age of the participants was 36.60 ± 10.10 years, and approximately 12596 (48.20%) were male. Significant differences were observed across the groups in terms of demographic characteristics (age, gender, race, marital status, education level, and family income), anthropometric and biochemical measures (BMI, TC, HDL-C, serum calcium, serum phosphorus, BUN, and UA), as well as health behaviors and conditions (alcohol use, hypertension, diabetes, CHD, stroke, and OA). The heavy exposure (serum cotinine level ≥3 ng/mL) group tended to be male, Non-Hispanic White, had a medium education level, living alone, had a low family income, had higher consumption of alcohol, and had higher incidence of hypertension, CHD, stroke, and OA.

**Table 1:** Baseline characteristics of participants in a cross-sectional study of tobacco smoke exposure and early-onset OA in the NHANES, 1999–2020 (N=26145)

*Characteristics*	*Total* *(N=26145)* *n (%)*	*Unexposed* *(N=11747)* *n (%)*	*Low exposure* *(N=6184)* *n (%)*	*Heavy exposure* *(N=8214)* *n (%)*	*p*
**Age** (years), mean ± SD	36.60 ± 10.10	37.50 ± 9.80	35.40 ± 10.30	36.20 ± 10.10	<0.001
**Gender**					<0.001
Male	12596 (48.20)	4758 (40.50)	2926 (47.30)	4912 (59.80)	
Female	13549 (51.80)	6989 (59.50)	3258 (52.70)	3302 (40.20)	
**Race**					<0.001
Non-Hispanic White	10815 (41.40)	4553 (38.80)	2267 (36.70)	3995 (48.60)	
Non-Hispanic Black	5401 (20.70)	1564 (13.30)	1707 (27.60)	2130 (25.90)	
Mexican American	4853 (18.60)	2857 (24.30)	1084 (17.50)	912 (11.10)	
Other	5076 (19.40)	2773 (23.60)	1126 (18.20)	1177 (14.30)	
**Education level** (years)					<0.001
<9	1715 (6.60)	847 (7.20)	411 (6.60)	457 (5.60)	
9–12	9493 (36.30)	2849 (24.30)	2400 (38.80)	4244 (51.70)	
>12	14937 (57.10)	8051 (68.50)	3373 (54.50)	3513 (42.80)	
**Marital status**					<0.001
Married/living with partner	16069 (61.50)	8282 (70.50)	3460 (56)	4327 (52.70)	
Living alone	10076 (38.50)	3465 (29.50)	2724 (44)	3887 (47.30)	
**PIR**					<0.001
Low income	8104 (31)	2571 (21.90)	2085 (33.7)	3448 (42)	
Medium income	9546 (36.50)	4174 (35.50)	2314 (37.4)	3058 (37.2)	
High income	8495 (32.50)	5002 (42.60)	1785 (28.9)	1708 (20.8)	
**Clinical measurements**					
TC (mmol/L)	5.00 ± 1.10	5.00 ± 1.00	5.00 ± 1.10	5.00 ± 1.1	<0.001
HDL-C (mmol/L)	1.40 ± 0.40	1.40 ± 0.40	1.30 ± 0.40	1.30 ± 0.40	<0.001
Serum calcium (mg/dL)	9.40 ± 0.40	9.30 ± 0.40	9.40 ± 0.40	9.40 ± 0.40	<0.001
Serum phosphorus (mg/dL)	3.70 ± 0.60	3.70 ± 0.60	3.70 ± 0.60	3.70 ± 0.60	0.01
BUN (mg/dL)	12.00 ± 4.30	12.30 ± 4.30	12 ± 4.30	11.50 ± 4.30	<0.001
UA (mg/dL)	5.20 ± 1.40	5.10 ± 1.40	5.40 ± 1.50	5.40 ± 1.40	<0.001
BMI (kg/m^2^)	29.00 ± 7.20	28.90 ± 6.90	30.10 ± 7.80	28.50 ± 7.10	<0.001
**Alcohol use**					<0.001
No	5739 (22)	3280 (27.90)	1569 (25.40)	890 (10.80)	
Yes	20406 (78)	8467 (72.10)	4615 (74.60)	7324 (89.20)	
**Hypertension**					<0.001
No	21147 (80.90)	9734 (82.90)	4959 (80.20)	6454 (78.60)	
Yes	4998 (19.10)	2013 (17.10)	1225 (19.80)	1760 (21.40)	
**Diabetes**					<0.001
No	24726 (94.60)	11097 (94.50)	5842 (94.50)	7787 (94.80)	
Yes	1419 (5.40)	650 (5.50)	342 (5.50)	427 (5.20)	
**CHD**					0.00
No	25953 (99.30)	11679 (99.40)	6143 (99.30)	8131 (99)	
Yes	192 (0.70)	68 (0.60)	41 (0.70)	83 (1)	
**Stroke**					<0.001
No	25863 (98.90)	11674 (99.40)	6126 (99.10)	8063 (98.20)	
Yes	282 (1.10)	73 (0.60)	58 (0.90)	151 (1.80)	
**OA**					<0.001
No	25059 (95.80)	11310 (96.30)	5968 (96.50)	7781 (94.70)	
Yes	1086 (4.20)	437 (3.70)	216 (3.50)	433 (5.30)	

PIR: poverty-to-income ratio. BMI: body mass index. TC: total cholesterol. HDL-C: high-density lipoprotein cholesterol. BUN: blood urea nitrogen. UA: uric acid. CHD: coronary heart disease. OA: osteoarthritis. All analyses were conducted using sample weights to ensure national representativeness.

### Univariable and multivariable logistic regression analyses

The univariable logistic regression analysis demonstrated that age, gender, race, education level, BMI, TC, serum calcium, BUN, UA, alcohol use, hypertension, diabetes, CHD, and stroke were associated with early-onset OA (Supplementary file Table 1). Univariable models showed that every unit increase in LN-transformed serum cotinine was associated with 5% higher odds of early-onset OA ( OR=1.05; 95% CI: 1.04–1.07, p <0.00).

Table 2 presents the results of the multivariable logistic regression analysis examining the relationship between serum cotinine levels and early-onset OA prevalence. After progressive adjustment (Model 4), the association remained robust: each unit increase in LN-cotinine corresponded to a 5% elevation in the odds of early-onset OA (AOR=1.05; 95 % CI: 1.04– 1.07). Participants in the heavy exposure category exhibited 1.52-fold higher odds of early-onset OA (AOR=1.52; 95% CI: 1.30–1.79) relative to the unexposed group, whereas low exposure (0.05–2.99 ng/mL) was not significantly associated (AOR=1.00; 95% CI: 0.83–1.19), confirming a dose–response relationship independent of sociodemographic, metabolic and comorbidity factors. A significant linear trend (p for trend <0.001) across cotinine categories further underscores the role of cumulative tobacco exposure in accelerating OA onset before the age of 55 years.

**Table 2:** Logistic multivariable regression analysis the association between tobacco smoke exposure and earlyonset OA across different exposure levels in the NHANES, 1999–2020 (N=26145)

*Variables*	*Model 1*		*Model 2*		*Model 3*		*Model 4*	
	*OR (95% CI)*	*p*	*AOR (95% CI)*	*p*	*AOR (95% CI)*	*p*	*AOR (95% CI)*	*p*
**LN cotinine** (ng/mL)	1.05 (1.04–1.07)	<0.001	1.05 (1.03–1.07)	<0.001	1.06 (1.05–1.08)	<0.001	1.05 (1.04–1.07)	<0.001
**Tobacco smoke exposure**								
Unexposed (Ref.)	1		1		1		1	
Low exposure	0.94 (0.79–1.11)	0.44	1.09 (0.92–1.3)	0.31	1.02 (0.85–1.21)	0.86	1 (0.83–1.19)	0.96
Heavy exposure	1.44 (1.26–1.65)	<0.001	1.54 (1.32–1.8)	<0.001	1.65 (1.41–1.93)	<0.001	1.52 (1.30–1.79)	<0.001
Trend test		<0.001		<0.001		<0.001	1.23 (1.14–1.34)	<0.001

Model 1: unadjusted. AOR: adjusted odds ratio. Model 2: adjusted for age, gender, race, education level, marital status, PIR. Model 3: adjusted as for Model 2 plus TC, HDL-C, serum calcium, serum phosphorus, BUN, UA, BMI. Model 4: adjusted as for Model 3 plus alcohol use, hypertension, diabetes, CHD, stroke. All analyses were conducted using sample weights to ensure national representativeness. LN: natural logarithm.

### Restricted cubic spline models

**Figure 2:**
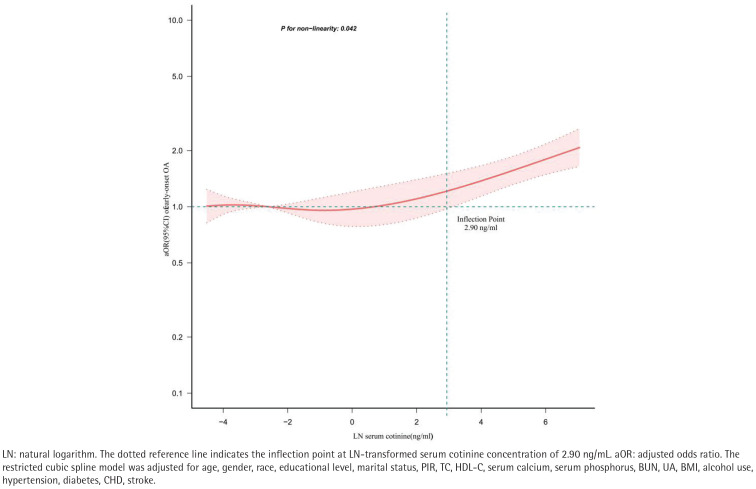
Restricted cubic spline analysis of the relationship between tobacco smoke exposure and early-onset OA prevalence in the NHANES, 1999–2020 (N=26145) LN: natural logarithm. The dotted reference line indicates the inflection point at LN-transformed serum cotinine concentration of 2.90 ng/mL. aOR: adjusted odds ratio. The restricted cubic spline model was adjusted for age, gender, race, educational level, marital status, PIR, TC, HDL-C, serum calcium, serum phosphorus, BUN, UA, BMI, alcohol use, hypertension, diabetes, CHD, stroke.

To illustrate the dose-response relationship between LN-transformed serum cotinine levels and earlyonset OA prevalence, a restricted cubic spline (RCS) regression model with multivariable adjustment was employed, as shown in Figure 2. A nonlinear positive association was observed between serum cotinine levels and the prevalence of early-onset OA (p for nonlinearity=0.04). Threshold effect analysis identified a critical point in this relationship at an LN-transformed serum cotinine concentration of 2.90 ng/mL. Beyond this value, a significant positive association was detected (OR=1.38; 95% CI: 1.17– 1.63, p=0.00), as detailed in Table 3.

**Table 3:** Inflection-point analysis of the association between tobacco smoke exposure and early-onset OA in the NHANES, 1999–2020 (N=26145)

*LN cotinine (ng/mL)*	*Adjusted*	*model*
	*AOR (95% CI)*	*p*
<2.90	1.02 (0.97–1.08)	0.35
≥2.90	1.38 (1.17–1.63)	0.00
Likelihood ratio test		0.00

AOR: adjusted odds ratio. Model adjusted for age, gender, race, education level, marital status, PIR, TC, HDL-C, serum calcium, serum phosphorus, BUN, UA, BMI, alcohol use, hypertension, diabetes, CHD, stroke. LN: natural logarithm.

### Subgroup analysis

**Figure 3:**
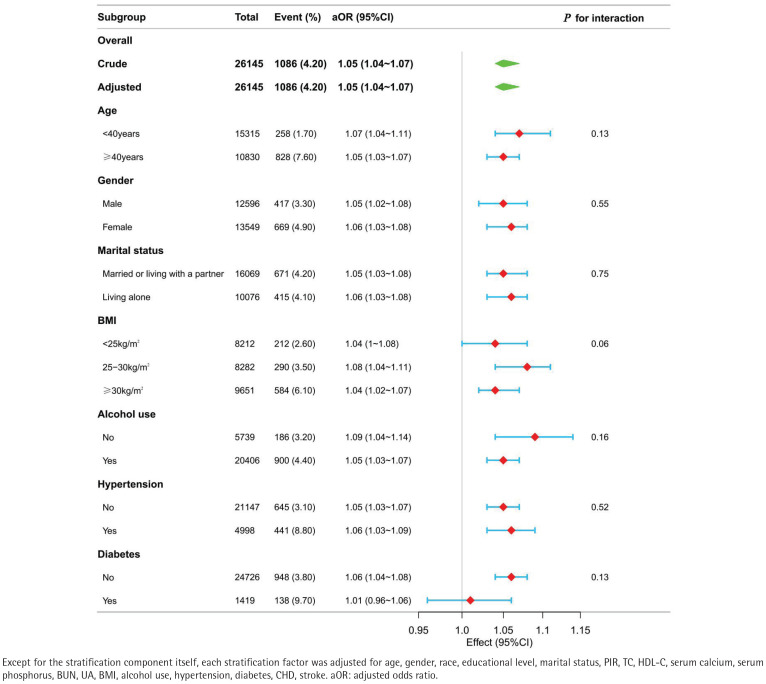
Forest plot of the association between tobacco smoke exposure and early-onset OA prevalence across subgroups in the NHANES, 1999–2020 (N=26145) Except for the stratification component itself, each stratification factor was adjusted for age, gender, race, educational level, marital status, PIR, TC, HDL-C, serum calcium, serum phosphorus, BUN, UA, BMI, alcohol use, hypertension, diabetes, CHD, stroke. aOR: adjusted odds ratio.

Subgroup analyses and interaction tests were conducted to evaluate whether the association between tobacco smoke exposure and early-onset OA remained consistent across various demographic and clinical subgroups (Supplementary file Table 2). Stratification factors included age, race, marital status, alcohol use, BMI, and the presence of chronic conditions including hypertension and diabetes. These analyses allowed for a detailed assessment of potential effect modification by these variables. As shown in Figure 3, the forest plot illustrates that interaction terms were tested at a significance level of p<0.05. No significant interaction effects were observed (all p>0.05), suggesting that the relationship did not differ substantially across the subgroups examined.

## DISCUSSION

This large cross-sectional study of individuals aged 20–54 years in the United States identified a nonlinear, positively saturated association between tobacco smoke exposure and early-onset OA. Specifically, when LN-transformed serum cotinine levels exceeded 2.90 ng/mL, each additional 1 ng/ mL increment in LN-transformed serum cotinine was associated with a 38% higher likelihood of developing early-onset OA.

Conversely, at concentrations below this threshold, no statistically significant association was observed between tobacco smoke exposure and the likelihood of early-onset OA. These findings indicate that elevated tobacco smoke exposure is significantly associated with an increased likelihood of early-onset OA, exhibiting a saturation effect beyond a certain concentration threshold. In addition, subgroup analyses further confirmed the robustness of this association. Employing Mendelian randomization, Xiao et al.^24^ provided genetic evidence that smoking behavior elevates the likelihood of developing osteoarthritis (OA). This is consistent with our conclusion that tobacco smoke exposure is associated with early-onset OA, highlighting the genetic predisposition that may underlie this association.

Previous research into the association between tobacco smoke exposure and OA have been sparse and uncertain. Some studies utilizing the NHANES database have reported a positive association between smoking and OA prevalence in the general US adults^25^. Moreover, a multicohort study incorporating both cross-sectional and longitudinal designs found no clear link between smoking and the incidence, prevalence, or progression of radiographic or symptomatic hip OA, either at baseline or over a follow-up period of four to five years^13^. Conversely, some studies have reported conflicting outcomes, suggesting that smoking might exert a protective effect on OA. For instance, a cross-sectional investigation found an inverse association between smoking and knee OA prevalence among older adults in Korea^26^. Similarly, a 2017 meta-analysis encompassing 34 independent observational studies indicated that cigarette smoking was linked to a decreased risk of knee OA, with a more pronounced association observed in men^10^. Despite the potential importance of this relationship, few studies have investigated the link between tobacco smoke exposure and early-onset OA with serum cotinine serving as an objective biomarker of exposure. Serum cotinine levels provide a reliable means of quantifying tobacco smoke exposure, thereby improving the generalizability and practical applicability of study outcomes. Given the crosssectional nature of NHANES data, which includes self-reported information, our findings suggest a potential association between tobacco smoke exposure and early-onset OA, highlighting the need for further longitudinal studies to establish causality. In contrast to prior research, this study specifically targets a younger demographic, comprising US participants aged 20–54 years. Serum cotinine levels were utilized to precisely quantify tobacco smoke exposure. The results indicated that below a specific threshold of LN-transformed serum cotinine, no significant association with early-onset OA was observed. Beyond this critical value, however, increasing levels were positively correlated with a higher prevalence of earlyonset OA. Further longitudinal or cohort studies are warranted to corroborate these findings and to further investigate this association.

The precise biological mechanisms through which smoking influences the development of OA are not yet fully elucidated. Several potential pathways have been proposed to explain how smoking might lead to OA. One plausible mechanism linking smoking to OA is oxidative stress, which results from the generation of reactive oxygen species by cigarette smoking, it can directly injure chondrocytes and suppress the synthesis of crucial extracellular-matrix components, ultimately driving cartilage breakdown^27^. Persistent oxidative stress imposed by cigarette smoke can outstrip the joint’s antioxidant capacity, sustaining a self-propagating loop of inflammatory activation and progressive tissue injury^28^. Using a three-dimensional human chondrocyte culture, Zamudio-Cuevas et al.^29^ recently demonstrated that cigarette smoke extract (CSE) triggers cytotoxicity, disturbs extracellularmatrix architecture, and heightens matrix metalloproteinase (MMP) activity through redox imbalance and IL-1β signaling while concurrently weakening antioxidant capacity, collectively indicating that smoking perturbs the delicate equilibrium of articular cartilage. Fernández-Torres et al.^28^ revealed that tobacco use amplifies cartilage injury by simultaneously heightening oxidative stress and arginase activity, especially in younger individuals. Elevated arginase, correlating with serum cotinine, drives profound osteoarthritic changes even when antioxidant counter-regulation is mobilized. In addition to these cellular and molecular mechanisms, smoking may worsen joint pathology through systemic effects such as vascular dysfunction^30^. Nicotineinduced vasoconstriction can diminish articular blood flow, compromising nutrient delivery and waste clearance. It may also impede the transport of immune mediators essential for tissue repair^31^. Given the observed association between tobacco smoke exposure and early-onset OA in this cross-sectional study, our findings suggest that reducing smoking and environmental nicotine exposure may be beneficial. Further high-level studies, such as prospective cohorts, are needed to establish causality and inform clinical guidelines and practice. Until such evidence is available, minimizing both active and passive smoking remains a prudent public health measure to potentially reduce the likelihood of early-onset OA.

### Strengths and limitations

The nationwide and multi-ethnic scope of our study enhances the applicability and generalizability of our findings. In addition, measuring serum cotinine as a biomarker of tobacco exposure markedly reduces misclassification and subjective bias, markedly strengthening the study’s validity. However, several limitations should be noted. First, the survey data focus primarily on the US population, and additional studies are required to verify these results in diverse geographical populations and settings. Due to its cross-sectional design, this study cannot infer causality between tobacco smoke exposure and early-onset OA. Furthermore, serum cotinine was measured only once, preventing assessment of long-term exposure dynamics. Although many confounding factors were adjusted for in this study, residual confounding may still exist, which could influence the results. One of the key limitations of our study is the lack of differentiation between primary and secondary exposure to tobacco smoke. Importantly, our analysis did not distinguish between primary (active) and secondary (passive) tobacco smoke exposure, which may lead to differential health impacts due to variations in concentration, duration, and biological effect. Active smoking involves direct inhalation of higher concentrations of harmful compounds, while passive exposure typically entails a lower dose environmental contact; these distinct pathways may differentially influence inflammatory, oxidative, and chondrodegenerative processes implicated in OA. Lastly, several measures relied on self-reported data, which are susceptible to recall or misclassification bias.

## CONCLUSIONS

This large, nationally representative study demonstrates a dose-dependent, non-linear association between tobacco smoke exposure and early-onset OA in adults aged <55 years. Serum cotinine levels ≥3 ng/mL were associated with a 52% increase in the odds of OA diagnosis before the age of 55 years. Together with the observed inflection point at 2.90 ng/mL, these data underscore the preventive potential of lowering population-level tobacco exposure. Future longitudinal and mechanistic studies are warranted to confirm causality and refine intervention strategies.

## Data Availability

The data supporting this research are available from the authors on reasonable request.
